# Snakebite victim profiles and treatment-seeking behaviors in two regions of Kenya: results from a health demographic surveillance system

**DOI:** 10.1186/s41182-022-00421-8

**Published:** 2022-04-29

**Authors:** Peter S. Larson, Morris Ndemwa, Aleksandra F. Thomas, Noriko Tamari, Paul Diela, Mwatasa Changoma, Abdullatif Mohamed, Miles C. Larson, Kaan Cem Ketenci, Kensuke Goto, Satoshi Kaneko

**Affiliations:** 1 Nagasaki University Institute of Tropical Medicine-Kenya Medical Research Institute (NUITM-KEMRI) Project, Kenya, Nagasaki, Nagasaki Japan; 2 Department of Epidemiology, University of Michigan School of Public Health, Ann Arbor, MI USA; 3 Kenya Medical Research Institute, Nairobi, Kenya; 4 Division of Health and Safety Sciences Education, Osaka Kyoiku University, Osaka, Japan; 5 Washtenaw Community College, Ann Arbor, MI USA; 6 University of Michigan Institute for Social Research, Ann Arbor, MI USA; 7 University of Michigan, Literature, Science and the Arts, Ann Arbor, MI USA

**Keywords:** Snakebite, Snake, Kenya, Health-seeking behaviors, Traditional treatments, Venomous animals, Epidemiology

## Abstract

**Introduction:**

Snakebites are a major cause of permanent injury and death among poor, rural populations in developing countries, including those in East Africa. This research characterizes snakebite incidence, risk factors, and subsequent health-seeking behaviors in two regions of Kenya using a mixed methods approach.

**Methods:**

As a part of regular activities of a health demographic surveillance system, household-level survey on snakebite incidence was conducted in two areas of Kenya: Kwale along the Kenyan Coast and Mbita on Lake Victoria. If someone in the home was reported to have been bitten in the 5 years previous to the visit, a survey instrument was administered. The survey gathered contextual information on the bite, treatment-seeking behavior and clinical manifestations. To obtain deeper, contextual information, respondents were also asked to narrate the bite incident, subsequent behavior and outcomes.

**Results:**

8775 and 9206 households were surveyed in Kwale and Mbita, respectively. Out of these, 453 (5.17%) and 92 (1.00%) households reported that at least one person had been bitten by a snake in the past 5 years. Deaths from snakebites were rare (4.04%), but patterns of treatment seeking varied. Treatment at formal care facilities were sought for 50.8% and at traditional healers for 53.3%. 18.4% sought treatment from both sources. Victims who delayed receiving treatment from a formal facility were more likely to have consulted a traditional healer (OR 8.8995% CI [3.83, 20.64]). Delays in treatment seeking were associated with significantly increased odds of having a severe outcome, including death, paralysis or loss of consciousness (OR 3.47 95% CI [1.56; 7.70]).

**Conclusion:**

Snakebite incidence and outcomes vary by region in Kenya, and treatment-seeking behaviors are complex. Work needs to be done to better characterize the spatial distribution of snakebite incidence in Kenya and efforts need to be made to ensure that victims have sufficient access to effective treatments to prevent death and serious injury.

**Supplementary Information:**

The online version contains supplementary material available at 10.1186/s41182-022-00421-8.

## Introduction

There are several millions of snakebite incidents each year, leading to over 1.8 million envenomings and up to 94,000 deaths [1]. Most bites occur in poor and undeveloped countries [2] and snakebite envenoming is inversely associated with the Human Development Index (HDI) [3]. The public health problem of snakebites has recently come to the forefront of international public health organizations after years of neglect [4, 5]. Accordingly, the World Health Organization (WHO) has added snakebites to their list of neglected tropical diseases (NTDs) [6, 7].

**Fig. 1:**
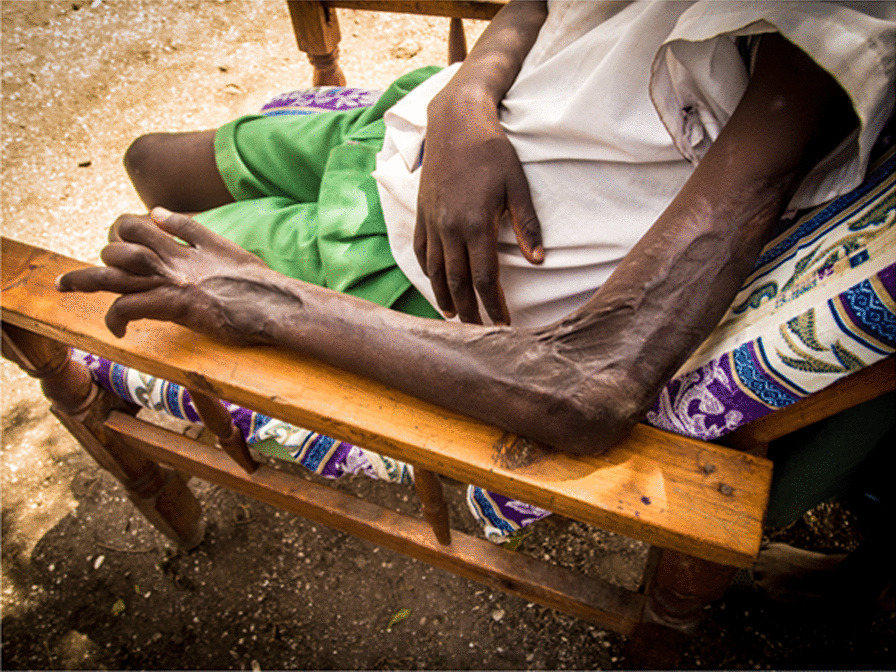
Permanent disfigurement from a puff adder bite, Kenya

In sub-Saharan Africa (SSA), a meta-analytic approach indicated that there are hundreds of thousands of bites yearly, of which thousands resulted in death or amputation [8]. Snakebites victims can come from urban or rural areas of SSA [9]. Rural and remote populations, specifically low-income agricultural workers, however, face the highest risk for bites and severe outcomes due to underlying environmental, social and infrastructural risk factors [10–12]. In Ghana, researchers estimated a 6% community prevalence of snakebites with a  3% case fatality rate [11]. Amputation and disability are common after snakebites [13] (see Fig. 1). Victims are often of the most economically active ages [14, 15]. Common locations of bites are on the feet or lower extremities [16]. Bites often occur while performing normal activities such as gathering wood or tending fields, though bites can occasionally occur within the home [17]. In the long term, snakebite risk may become exacerbated in SSA due to climate change [18] further impacting rural communities in SSA.

Treatment options for snakebites in SSA are limited even when victims seek help at formal facilities [19]. Antivenoms, given their high cost, are often unavailable to both individuals and struggling health system; lack of access to antivenoms, therefore, is a major public health issue in SSA [20–23]. Victims must frequently travel long distances, often at great expense, to receive care at large referral hospitals [14]. Traditional healing practices are then sometimes the first choice for snake bite victims in SSA [24, 25]. Types of herbal treatments vary by region and ethnic group [26–29] even within one of the regions chosen for this study [27]. Given the common nature of bites, because of the association of snakes with various mystical and malevolent forces and because of the relatively high costs of medical care in resource poor contexts, traditional practitioners have made snakebites a major part of their practice [30].

Treatment delays can worsen prognosis and result in high medical care expenses to victims and health systems [14, 31–33]. Prompt formal treatment for snakebites, particularly in rural areas, can reduce mortality and severe injury [34–36]. However, up to 80% of snakebite victims in Kenya consult traditional healers before seeking services from a formal health facility [15, 37, 38]. Anecdotal reports from health care workers in Kitui also confirm delays in treatment seeking from consultation with traditional healers [39]. However, the interplay of traditional and formal methods of treatment for snakebites in the regions of study and the exact influence that competing sources of treatment have on the timing of care and clinical manifestations is unclear. Of interest to this research is the exact nature of treatment-seeking behavior among snakebite victims in rural areas of Kenya and how choices of immediate treatment might impact short and long-term clinical manifestations.

This research uses a combination of structured surveys and participant recited stories of bite incidents to explore possible determinants of snakebites and treatment-seeking behavior in two rural areas of Kenya. We test three main hypotheses. First, bite risk, demographics of bite victims and specific conditions of the bite incident will differ between regions. Second, treatment strategies given a snakebite will vary between individuals, including a mix of traditional and formal treatments. Third, the choice of treatment type and the timing of treatment will impact clinical manifestations, such as death or severe injury.

### Snakes species in Kenya

It is estimated that there are 34 genera and 158 species of snakes native to the East Africa regions [40]. Kenya is home to numerous species of venomous and medically important snakes [38]. Behavior and habitats vary by snake species and create conditions of bite risk that are unique to each [41]. There are several elapid species in Kenya including the black mamba (*Dendroaspis polylepis*), the eastern green mambas (*Dendroaspis angusticeps*), eastern Jameson’s mamba (*Dendroaspis jamesoni*), as well as cobras, such as the black-necked spitting cobra (*Naja nigricollis*), the large brown spitting cobra (Naja ashei), the forest cobra (*Naja melanoleuca*) and the Egyptian cobra (*Naja haje*). Vipers include the extremely dangerous puff adder (*Bitis arietans*), the mole viper (*Atractaspis sp.* and the night adder (*Causus sp.*). The colubrid boomslang (Colubridae) (*Dispholidus typus*) is also commonly found in Kenya. Venoms in medically important snakes indigenous to Kenya include neurotoxic venoms typical of elapids, hemotoxic venoms of boomslangs, and the deadly cytotoxic venom of puff adders [42]. This study was conducted in areas in both the Coastal and Lake regions of Kenya. Black mambas, puff adders, black-necked and large brown spitting cobras, mole vipers and night adders and boomslangs are found in both coastal and lake areas. Eastern green mambas are only found on the coastal region. Egyptian cobras are absent from the coastal region [38]. Researchers confirmed through conversations with hospital staff that polyspecific antivenom [(Afriven (ASVS) 10] [23] is at least nominally available at health facilities in both of the study regions included in this study.

## Methods

### Data

**Fig. 2:**
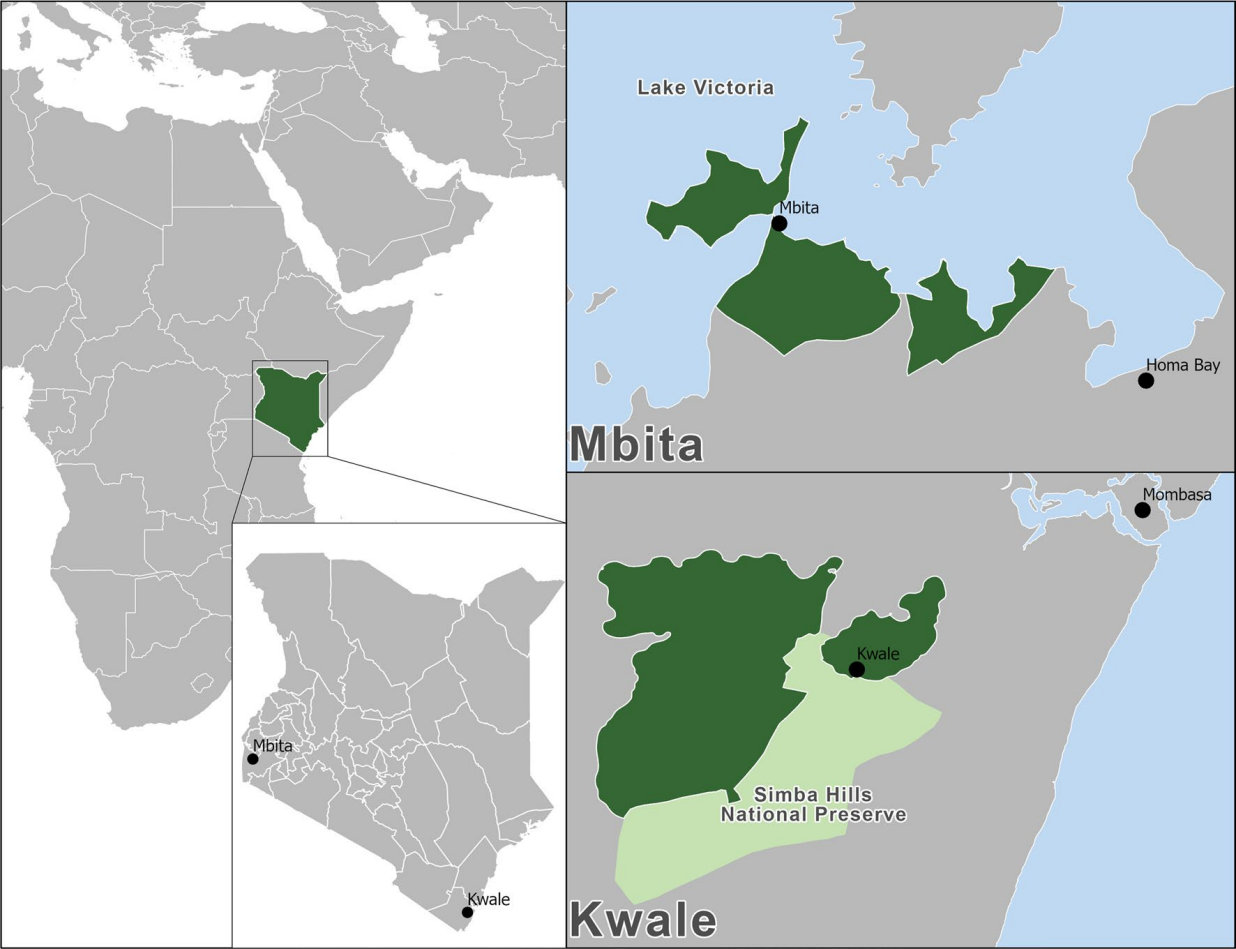
Map of Kenya and study areas

The Nagasaki University Institute of Tropical Medicine (NUITM) in cooperation with the Kenya Medical Research Institute (KEMRI) maintains a Health Demographic Surveillance System (HDSS) in two areas of Kenya: Mbita in Homa Bay County along the shores of Lake Victoria and Kwale County along the Kenyan Coast (see Fig. 2). Covering a population of more than 50,000 people in both areas, the HDSS regularly collects information on births, deaths, migrations, and pregnancies through direct home visits by locally staffed field enumerators. The HDSS also records data on household demographics, assets, economic activities, and major health events. Survey activities are conducted with the consent of the community and individuals. All households within the geographic bounds of the HDSS are eligible for inclusion in all regular HDSS activities. The population-based nature of the HDSS means that data for nearly all households are recorded on a quarterly basis. A full description of the HDSS and its procedures can be found in [43].

During a survey round starting in early 2016, the HDSS added a question asking household representatives if any household member had experienced a snakebite in the past 2 years. If the household representative reported that a family member had been bitten in the past, an attempt was made to speak with the victim and obtain specific information on the victim, bite timing, part of the body bitten, and outcomes. If the victim was not available or was deceased, information was obtained using the household head as a proxy.

### Household-level determinants of snakebites

As poverty has been found to be associated with snakebites risk is other contexts, we tested for associations between SES and snakebite risk at that household-level using Chi-square tests.

Distance to treatment sources has been recognized by the WHO as a [44] is a possible barrier to prompt treatment and thus a possible risk factor for more serious clinical manifestations. Research on the impact of distance to treatment seeking for snakebites and outcomes are scarce in the published literature. Limited evidence has suggested that distance to care may be an important determinant both in [45] and outside of African contexts [46, 47]. Latitude and longitude locations of households are recorded for all homes withing the HDSS. We used the GPS location of the household to calculate the Euclidean (straight-line) distance to the nearest primary and tertiary health facilities for all households and compared distances using t-tests of means.

### Qualitative information on bite incidents

To confirm responses collected within the survey, participants were asked to narrate the conditions surrounding the bite, the bite location, subsequent treatment seeking and long-term outcomes of the bite. Field workers recorded participant stories in English on tablet computers as a part of the regular survey process. Stories were then examined by the research team for keywords and subsequently classified into themes such as traditional practices, use of formal health facilities, length of hospital stay, injuries, and long-term outcomes including death. Responses were then coded for frequency of particular responses and checked for agreement with survey data.

### Statistical methods

Descriptive statistics were produced for the entire survey sample and by region. *T*-tests were used to compare means between regional and snakebite groups for continuous variables. Chi-square tests were performed to assess independence of categorical variables with outcomes. Logistic regression was used to test associations between determinants and the presence or absence of snakebite.

## Results

### Household characteristics and snakebites by region

8756 households were surveyed in Kwale, and 9174 were surveyed in Mbita. Out of these, 453 (5.2%) and 92 (1.0%) households (approximate incidence 103.47 and 100.28 households per year per 10,000 households, respectively), respectively, reported that at least one person had been bitten by a snake in the past 5 years. Households in Mbita were 1.92 kms away from the nearest health facility and 10.10 kms away from a tertiary care facility. Households in Kwale were 3.48 kms away from the nearest health facility of any kind and 7.17 kms away from a tertiary care facility. Household-level socio-economic status (SES) data were available for a subset of homes. SES was not a determinant of bite incidence in Mbita, but was weakly associated (*p* = 0.056) with bites in Kwale. In Kwale, households that reported bites were located significantly farther away from a health facility than households that did not (4.73 km vs 3.41 km, *p*< 0.0001). Access to tertiary care was similar in Kwale for both households that did and did not report bites (7.20 km vs. 7.17 km, *p* = 0.844.) In Mbita, however, households of bite victims were located farther away from tertiary care than other households (11.2 km vs. 10.1 km, *p* = 0.027); see Table 1.

**Table 1:** Descriptive table of household-level determinants of snakebites to illustrate differences in association of SES and snakebites and access to health care resources by region

	Kwale				Mbita			
	[ALL]	No bite	Bite	*p*	[ALL]	No bite	Bite	*p*
	*N* = 8756	*N* = 8303	*N* = 453		*N* = 9174	*N* = 9082	*N* = 92	
SES_quants								
Least poor	1189 (20.4%)	1158 (20.6%)	31 (15.0%)	0.056	1477 (16.8%)	1461 (16.8%)	16 (18.2%)	0.874
2	1179 (20.2%)	1132 (20.1%)	47 (22.8%)		1713 (19.5%)	1694 (19.4%)	19 (21.6%)	
3	1098 (18.8%)	1067 (19.0%)	31 (15.0%)		1853 (21.0%)	1833 (21.0%)	20 (22.7%)	
4	1177 (20.2%)	1123 (19.9%)	54 (26.2%)		1759 (20.0%)	1745 (20.0%)	14 (15.9%)	
Poorest	1193 (20.4%)	1150 (20.4%)	43 (20.9%)		2004 (22.8%)	1985 (22.8%)	19 (21.6%)	
Distance to health facility (kms)	3.48 (2.07)	3.41 (2.06)	4.73 (1.71)	<0.001	1.92 (1.16)	1.92 (1.16)	1.70 (1.15)	0.063
Distance to tertiary facility (kms)	7.17 (4.56)	7.17 (4.62)	7.20 (3.11)	0.844	10.1 (4.33)	10.1 (4.33)	11.2 (4.91)	0.027

Results for SES are counts and percentages. Means and standard deviations for distance measures are presented for continuous variables

### Snakebite victim profiles and bite context

We did not find evidence to suggest that gender was associated with bites in either region. Ages of snakebite victims ranged from 1 to 82 years of age (median: 30 years, mean 33.18 years, IQR: 18–45.) (see distribution of ages in Additional file 1: Fig. S1). Most people were bitten on the foot or leg (91.9%) followed by the arm or hand (6.6%) (Fig 1) but there were regional differences. In both areas, more victims reported being bitten in the dry season than in the rainy season (66.67% vs. 33.33%). Most people were bitten in the fields around the home or in the compound, but many people reported having been bitten inside the home. Oral histories collected during data collection indicated that many people were bitten while doing agricultural activities or collecting firewood. Most bites occurred in fields located close to the home (33.6%) or within the housing compound (25.5%). Some bites occurred indoors (14.6%). Among the 203 respondents speaking on their own behalf, 78 (38.4%) reported not having seen the snake at all (not shown in table). See Table 2 for full results.

**Table 2:** Victim profiles and bite conditions, both overall and by region

	[ALL]	Kwale	Mbita	*p*
	*N* = 396	*N* = 315	*N* = 81	
Respondent				
Self-reported	203 (51.3%)	154 (48.9%)	49 (60.5%)	0.082
Proxy	193 (48.7%)	161 (51.1%)	32 (39.5%)	
Gender				
Female	188 (47.5%)	153 (48.6%)	35 (43.2%)	0.461
Male	208 (52.5%)	162 (51.4%)	46 (56.8%)	
Age of respondent	33.2 (18.7)	33.9 (19.0)	30.5 (17.4)	0.136
Location of bite				
Foot or leg	362 (91.9%)	292 (93.3%)	70 (86.4%)	0.039
Arm or hand	26 (6.60%)	15 (4.79%)	11 (13.6%)	
Head/neck/face	3 (0.76%)	3 (0.96%)	0 (0.00%)	
Torso	3 (0.76%)	3 (0.96%)	0 (0.00%)	
Season				
Dry	262 (66.7%)	214 (67.9%)	48 (61.5%)	0.348
Rainy	131 (33.3%)	101 (32.1%)	30 (38.5%)	
Location of incident				
In the field surrounding the house	133 (33.6%)	99 (31.4%)	34 (42.0%)	0.036
In the compound	101 (25.5%)	84 (26.7%)	17 (21.0%)	
Away from home or compound but outside	61 (15.4%)	52 (16.5%)	9 (11.1%)	
Inside the house	58 (14.6%)	41 (13.0%)	17 (21.0%)	
Away from home or compound but inside	43 (10.9%)	39 (12.4%)	4 (4.94%)	

*p*-values represent Chi-square tests for independence between region with categorical variables and* T*-tests for the continuous measure of age

### Care-seeking behavior and clinical manifestations

Half (50.8%) of victims visited a formal health facility and (53.8%) of victims received treatment from a traditional healer following the bite. 18.7% of victims consulted both types of treatment sources. Approximately 32.1% and 35.1% of victims received care only from the formal facility and traditional healer, respectively. 14.1% of victims reported not seeking treatment at all. Most people who were treated at a formal health facility presented on the day of the bite (77.5%). Respondents could report more than one clinical manifestation of bites. These included vomiting (17.8%), loss of consciousness (10.9%), paralysis (0.51%), scarring and skin injuries (13.2%), and permanent injury defined as long-term debility in movement and/or ability to perform daily activities (11.0%). Only 16 (4.04%) victims died as a result of the bite. See Table 3 for full results.

Respondents were asked what kinds of traditional treatments they received, regardless of whether they reported visiting a traditional healer. 173 (48%) mentioned that they received “herbs”. 172 (47.8%) people said they had a snake stone applied. This included 79 people who visited formal health facilities for treatment, 58 of whom who had not explicitly reported having gone to a traditional healer. 126 (35.0%) reported receiving some form of a cut, suck, and bind treatment (e.g., “saroo” in Luo or “finywa chanja”). (Results not shown in table.)

**Table 3:** Treatment-seeking patterns and clinical manifestations overall and by region

	[ALL]	Kwale	Mbita	*p*
	*N* = 396	*N* = 315	*N* = 81	
Treated at formal facility	201 (50.8%)	158 (50.2%)	43 (53.1%)	0.730
Treated by traditional healer	213 (53.8%)	159 (50.5%)	54 (66.7%)	0.013
Formal treatment only	127 (32.1%)	109 (34.6%)	18 (22.2%)	0.046
Traditional healer only	139 (35.1%)	110 (34.9%)	29 (35.8%)	0.986
Both formal and traditional	74 (18.7%)	49 (15.6%)	25 (30.9%)	0.003
Did not seek treatment	56 (14.1%)	47 (14.9%)	9 (11.1%)	0.485
Length of time until formal treatment				
Same day	155 (77.5%)	127 (80.4%)	28 (66.7%)	0.035
Following day	35 (17.5%)	22 (13.9%)	13 (31.0%)	
2–3 days after the bite	7 (3.50%)	7 (4.43%)	0 (0.00%)	
Within a week later	3 (1.50%)	2 (1.27%)	1 (2.38%)	
Clinical manifestations				
Vomiting	70 (17.8%)	39 (12.4%)	31 (39.2%)	< 0.001
Loss of consciousness	43 (10.9%)	22 (7.01%)	21 (26.6%)	< 0.001
Paralysis	2 (0.51%)	1 (0.32%)	1 (1.23%)	0.368
Scarring	52 (13.2%)	33 (10.5%)	19 (24.1%)	0.003
Permanent physical debilitation	42 (11.0%)	14 (4.59%)	28 (35.9%)	< 0.001
Died	16 (4.04%)	11 (3.49%)	5 (6.17%)	0.338

*p*-values are from Chi-square tests for Independence between region and categorical variables

**Fig. 3:**
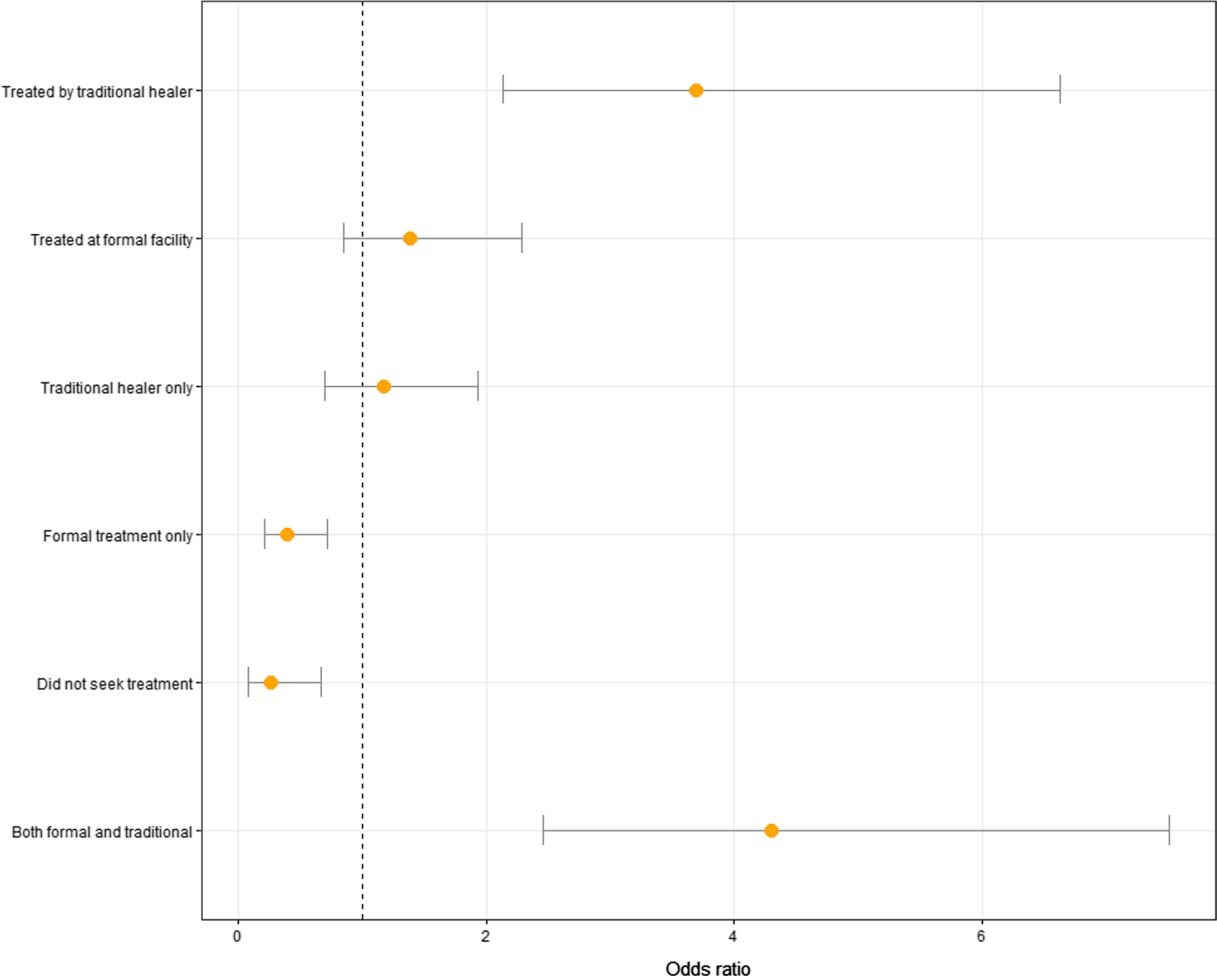
Odds ratios and confidence intervals from bivariate logistic regression models of severe outcomes (defined as death, paralysis, permanent disability or loss of consciousness) and various treatment strategies

**Fig. 4:**
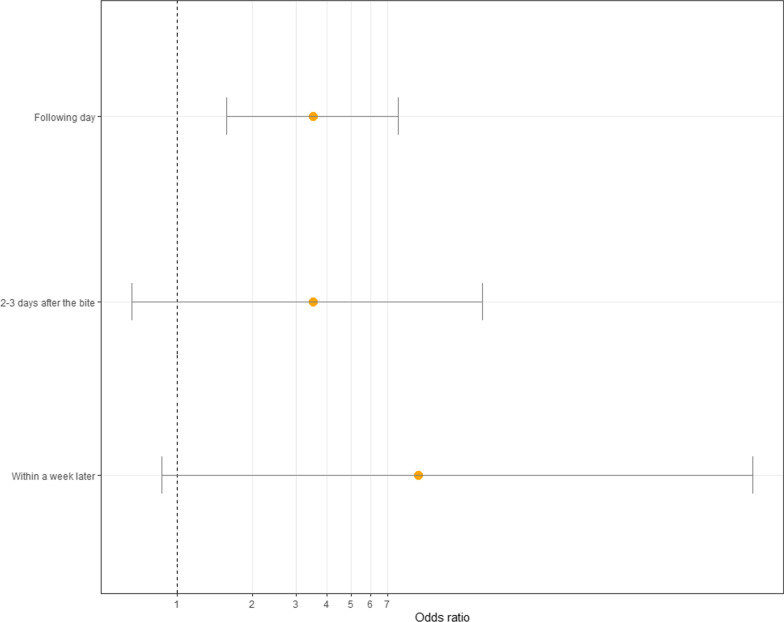
Odds ratios and confidence intervals from a logistic regression models of severe outcomes (defined as death, paralysis, permanent disability or loss of consciousness) and time to treatment. Reference is same day treatment

We defined a severe outcome as any bite that resulted in death, paralysis, loss of consciousness, or reported permanent physical debilitation. Among the 392 persons for whom outcome information was available, 82 (20.1%) were found to have severe outcomes. There was no significant difference in outcome for those who sought treatment at formal facilities (OR 1.39 95% CI [0.85; 2.29]). Bites treated by traditional healers were more severe (OR 3.69 95%CI [2.14; 6.63]) but there was no significant difference in outcomes for those who sought treatment only from traditional sources (OR 1.17 95% CI [0.70; 1.93]). Seeking treatment from both sources was associated with increased odds of having a severe outcome (OR 4.30 95% CI [2.46; 7.51]). The odds of having a severe bite was significantly lower for those who did not seek any type of treatment (OR 4.30 95% CI [2.46; 7.51]). Delaying treatment from formal facilities until the following day was associated with significantly increased odds of having a severe outcome (OR 3.47 95% CI [1.56; 7.70]). See Table 4 for full results and Fig. 3 and 4.

Though we were not able to determine which treatment resources were utilized first, we tested whether visiting a traditional healer was associated with delays in seeking treatment from a formal health clinic. Among those who did visit a formal facility, the odds of having seen a traditional healer was significantly higher for those people who visited a clinic the next day (OR 8.89[95% CI 3.83, 20.64]). The odds were even higher for those who waited 2 to 3 days after the bite 18.47[95% CI 1.04, 76.24]). (Results not shown in table.)

**Table 4:** Health-seeking behaviors and bite severity

	[ALL]	Minor outcome	Severe outcome	OR [95% CI]	*p*
	*N* = 392	*N* = 310	*N* = 82		
Treated at formal facility	199 (50.8%)	152 (49.0%)	47 (57.3%)	1.39 [0.85; 2.29]	0.185
Treated by traditional healer	209 (53.3%)	146 (47.1%)	63 (76.8%)	3.69 [2.14; 6.63]	<0.001
Formal treatment only	127 (32.4%)	112 (36.1%)	15 (18.3%)	0.40 [0.21; 0.72]	0.002
Traditional healer only	137 (34.9%)	106 (34.2%)	31 (37.8%)	1.17 [0.70; 1.93]	0.542
Both formal and traditional	72 (18.4%)	40 (12.9%)	32 (39.0%)	4.30 [2.46; 7.51]	<0.001
Did not seek treatment	56 (14.3%)	52 (16.8%)	4 (4.88%)	0.26 [0.08; 0.67]	0.003
Length of time post-bite					
Same day	153 (77.3%)	126 (83.4%)	27 (57.4%)	Ref.	Ref.
Following day	35 (17.7%)	20 (13.2%)	15 (31.9%)	3.47 [1.56; 7.70]	0.003
2–3 days after the bite	7 (3.54%)	4 (2.65%)	3 (6.38%)	3.50 [0.61; 17.7]	0.146
Within a week later	3 (1.52%)	1 (0.66%)	2 (4.26%)	8.61 [0.67; 277]	0.095

Any bite which resulted in paralysis, permanent injury or death was considered to be a case of severe envenoming (“severe outcome”). Bite severity was also assessed for gender, age and reported snake species

### Bite incident narratives

To obtain more detailed contextual information around the bite and clinical manifestation, victims or respondent representatives were asked to narrate the conditions leading up to, during, and after the incident. Each interview was conducted in Swahili and transcribed in English by the field data collector. We present relevant oral histories of the bite incident in three parts: conditions of the bite incident, treatment seeking, and long-term outcomes.

#### Treatment seeking from formal facilities

Some victims sought immediate treatment from formal health care providers:

“He was opening his field door to let his animals graze when he was bitten by a snake on his leg. He tied his leg up with a piece of cloth and went to the hospital. He recovered within a week.”

“She was on her way back home from school when she was bitten by a snake. She was taken to the nearest hospital for medication. Two days later she recovered.”

#### Treatment seeking from traditional healers

Many individuals related experiences of visiting traditional healers following the bite. While it is impossible to determine whether the outcomes are associated with visiting the traditional healers, the stories provided supporting evidence for data collected through the survey instrument.

Some victims sought care from traditional healers and suffered lengthy recovery times. One woman was bitten by what she claimed was a green mamba:

“He tied a string around her leg and took her to a traditional healer for treatment. It took 2 months for the woman to recover.”

Some people sought out traditional healers after visiting a health facility. The reasons for this were unclear:

“He was taken to the hospital for treatment. After that he went to a traditional healer.”

“He was at his friend’s house studying when a snake bit him. His friend came to help and they saw a puff adder; the friend killed the snake. The mother came and tied a string around his leg, then took him to a traditional healer for treatment. It took one month of recovery before the boy could return to school.”

Others visited traditional healers exclusively and suffered minor, long-term effects. It is unclear whether the long-term impacts are due to delayed treatment or were avoidable if given formal care.

“He went to a traditional healer for treatment. He still sometimes feels pain or as if something is touching the affected area.”

“He placed a snake stone on the affected area and went to a traditional healer. He still sometimes feels pain and as if something is crawling up his leg.”

#### Self-treatment

Though we did not include a question on our survey for self-treatment, we found that many victims did, in fact, attempt to treat themselves. The narratives exclusively indicate self-treatment by traditional means.

“He went home and scratched the affected part and removed two teeth. He then treated himself with a snake stone.”

“Her mom went home and brought back a snake stone to place on the affected area to absorb the poison.”

Self-treatment sometimes predicated receiving care from a traditional healer:

“He came back home and placed a snake stone on the affected area, then was taken to a traditional herbalist for treatment.”

“He applied paraffin and a snake stone to absorb the venom. The following day he went to a traditional healer.”

“He ran home and placed a snake stone on the affected area, then he received herbal treatment from a traditional healer. A few hours later the snake stone fell off. Six hours later he recovered to his normal condition.”

“He was around his home compound when he was bitten. After a few minutes his whole leg was swelling. He treated himself with a snake stone then went to the hospital the following day. He was admitted for two months then discharged.”

#### Mixed treatment and delayed treatment

Some victims indicated that they received both formal and traditional treatments, suggesting that treatment sources in these regions are not mutually exclusive. One individual received treatment from his parents while in the hospital:

“Once home, he started vomiting and explained what happened to his parents. They took him to the hospital. In the hospital during treatment his parents placed a snake stone to absorb the venom. He was admitted for 23 days.”

One respondent, married to a traditional healer, provided a narrative for receiving both types of care and also an indication of treatment delay:

“She went back to the house and tied a string around her ankle then called her husband, who is a traditional doctor. He pulled out the snake’s teeth with a snake stone and the following day she went to the hospital where she was injected with antivenom and was treated.”

“He was playing in the house when a snake bit his hand. He started crying and his hand swelled up immediately. He was taken to a traditional doctor for treatment then was taken to the hospital. He was injected with antivenom at the hospital and recovered within 1 week.”

Another person waited 3 days before seeking hospital treatment and recovered within weeks:

“He was crossing the road to a nearby farm field when he heard a strange sound and saw a black mamba. The snake attacked him and managed to bite his leg. He tied a string around his leg and went to a traditional healer. After 3 days he went to the hospital and was injected with antivenom; he was admitted for 3 weeks.”

One individual first attempted to self-treat the wound, then sought care from a traditional healer. However, the victim’s condition worsened, he was eventually admitted to a hospital.

“He came back home and place snake stone on the affected area, then was taken to a traditional herbalist for treatment. His condition worsened, and after 1 week he was taken to the hospital and was admitted for two months.”

“Her father came and rushed her to a nearby traditional doctor who gave her some herbs. Her body became swollen and she was taken to Kisumu for treatment after 1 week.”

“He was sleeping at night when he felt a painful sting on his hand. He started bleeding profusely. His brother called their mother, who came and tied a string around his hand. She took him to a traditional healer who applied medicine to the affected area. He was taken to the hospital the following day and was given antivenom. He was transferred to Homa Bay Referral Hospital where he was admitted for 1 year.”

“She was taken to a traditional healer. After a week she was taken to the hospital for further treatment; she was admitted for ten months. She now has a permanent scar on her foot.”

“He came home and immediately went to the nearby traditional healer. His condition worsened, so he went to the hospital. It took 2 years in the hospital to recover from the incident.”

Finally, while not a case of delayed treatment, we encountered a story of an infant being bitten in the home. Despite being taken to a facility and given treatment, the infant died soon after:

“A woman had gone to her farm field and brought her baby with her. She placed the baby under a mango tree so she could tend to the field. A snake attacked and bit the baby’s hand, so the mother grabbed her baby and ran home. She placed a snake stone on the affected area and drove to the hospital. The hospital injected the baby with antivenom, but it was too late. After the baby died, the mother took her body and returned home to give her a proper burial.”

## Discussion

Using a comprehensive demographic surveillance system, we have shown prevalences of snakebite victims in two regions of Kenya, for which such data were previously unavailable. We have also shown wide variation in the profiles of victims, treatment-seeking behaviors, and clinical manifestations both within and between distinct regions of Kenya. The overall prevalence of bites, the risk of death or serious injury, the context of the bite incident, and the bite area on the body agree with other studies on snakebite incidence in Kenya and similar regions in Africa [14, 48, 49]. We also found that treatment-seeking behavior in these two regions of Kenya follows the complex spectrum of behaviors seen in other areas of the world.

We found that half of respondents sought the assistance of a traditional healer, an underestimate compared with results from other studies [15, 49]. On the other hand, half of the victims surveyed indicated utilizing formal health care to treat bites agreeing with results from other studies [49]. Some research has shown that a community-level lack of confidence in health workers in terms of procedural knowledge and treatment administration leads many community members to seek treatment from traditional healers [50, 51]. On the other hand, it is possible that some victims might hold positive attitudes toward formal clinics leading them to seek treatment in formal clinics first. Our study was not designed to collect specific quantitative data on factors which contribute to choice of treatment sources, but bite narratives indicated that there are diverging attitudes toward these two types of care.

We found that many people in this region utilize both traditional and formal health care to treat bites. During the bite narratives, we found that some respondents reported visiting traditional healers after seeking care at a clinic, rather than before, with one respondent even receiving traditional care while hospitalized. This could indicate that some people in these communities view the two sources of care not as mutually exclusive, but complementary. This would assume that one could receive effective treatment upon presentation at a clinic, but practical shortfalls or cultural demands might lead patients to supplement care through the use of traditional healers or through self treatment using traditional methods. We note that several victims reported self-treatment with snake stones before visiting a clinic.

Our results indicate that use of traditional healers was associated with delays in receiving treatment from formal sources. While many snakebites are not serious, even short delays in treatment can lead to worse outcomes [52, 53]. Coordination between healers and formal health facilities could help insure that severe bites receive prompt and effective treatment [54]. Other studies in similar contexts have noted that snakebite patients use herbal treatments before presenting to a hospital in Nigeria [49]. Delays in formal treatment seeking after seeking treatment from traditional healers have been noted elsewhere [25].

We found that the qualitative information collected through the bite narratives was an unexpected strength of our research. Initially, we viewed the narratives as a means to ensure data quality and account for possible recording errors by field staff. We quickly learned that the experiences of snakebites vary widely among individuals, both before and at the time of bite, and in terms of the long-term outcomes. We thus found the narratives an essential tool for understanding the lived experience of snakebite victims. Future research efforts should take more formal approaches to the qualitative study of snakebites in these particular regions of Kenya.

There were many limitations to this research. First, the self reported nature of the measure complicates the ability to recall specific conditions around the bite and long-term outcomes. Future work might create a snakebite registry in cooperation with local partners, where bites are recorded as they happen, while information is still fresh. This would allow the use of the traditional snakebite severity score to more formally assess the level and amount of envenomation of victims [55]. Next, when testing for associations of bite severity and treatment-seeking behavior, the direction of causality was obscure. It was difficult to determine if specific outcomes led people to seek particular types of treatment or whether particular types of treatment were associated with specific outcomes.

Another limitation of our work was the exclusive focus on physical outcomes. Future work may work to assess the association between bites, choices of treatments and long-term psychological health as has been suggested in other studies [39, 56, 57]. To date, however, almost no work has been done on psychological outcomes in SSA outside of two studies in Nigeria [58]. One found that having snakebite related physical symptoms and having been bitten in the past were both strongly associated with mild and severe depression [59]. Another found that bite victims were at increased risk for symptoms of post-traumatic distress syndrome and poorer quality of life [60]. While we did not collect detailed information on the psychological well-being of participants, the qualitative narratives indicate that the bite incident remains a major life event suggesting that it may be a salient part of understanding the true impacts of snakebites.

## Supplementary Information

**Additional file 1: Figure S1.** Distribution of ages of snakebite victims.

## Data Availability

Data are available upon reasonable request. All codes used for this research are available at the GitHub repository https://github.com/kambanane/Snakes.

## Abbreviations

HDI: Human Development Index
NTD: Neglected tropical disease
CI: Confidence interval
WHO: World Health Organization
SSA: Sub-Saharan Africa
SES: Socio-economic status
