# Supplementary figures and images for: Snakebite victim profiles and treatment-seeking behaviors in two regions of Kenya: results from a health demographic surveillance system
Source: Trop Med Health. 2022 Apr 29;50:31. doi: 10.1186/s41182-022-00421-8 (PMC9052588; doi:10.1186/s41182-022-00421-8)

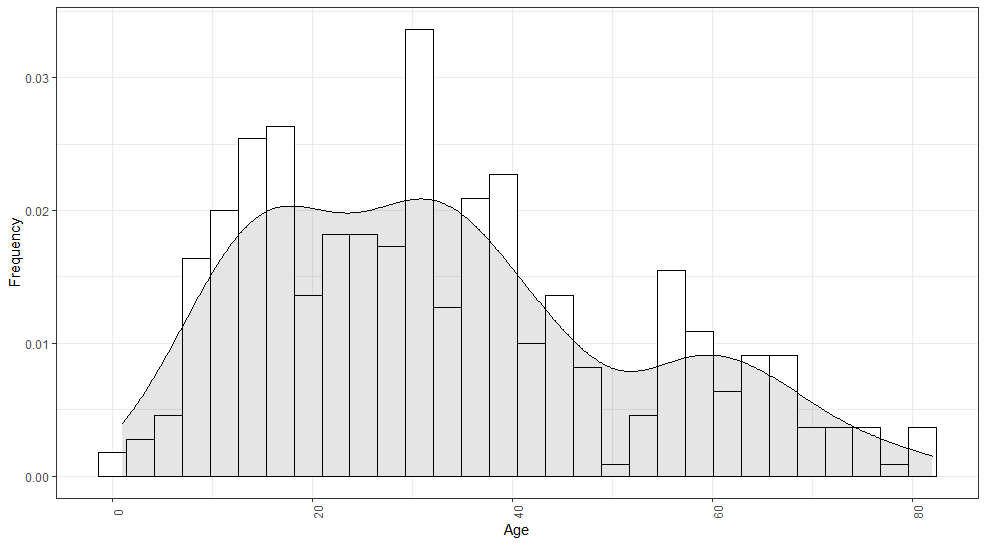

Supplement: Supplementary file 1 — Additional file 1: Figure S1. Distribution of ages of snakebite victims. [file 41182_2022_421_MOESM1_ESM.png]
